# Is the Middle Meningeal Artery the Optimal Path for Dural Arteriovenous Fistula Embolization?

**DOI:** 10.3389/fneur.2021.675355

**Published:** 2021-05-31

**Authors:** Han Su, Kan Xu, Yiheng Wang, Jinlu Yu

**Affiliations:** Department of Neurosurgery, The First Hospital of Jilin University, Changchun, China

**Keywords:** middle meningeal artery, dural arteriovenous fistula, endovascular treatment, classification, prognosis

## Abstract

**Background:** The middle meningeal artery (MMA) is the optimal arterial path for endovascular treatment (EVT) of dural arteriovenous fistulas (DAVFs). However, the details are not completely understood.

**Materials and Methods:** We performed a retrospective study of patients who were admitted to the First Hospital of Jilin University with a diagnosis of cranial DAVF with involvement of the MMA as a feeding artery. On the basis of the arterial path chosen and the role of the MMA in the first EVT procedure, EVT was divided into three types (I–III), each of which was further divided into two subclasses (a and b). The degree of embolization was analyzed.

**Result:** The 104 included patients ranged in age from 13 to 80 years (mean, 53.6 ± 11.8 years). There were 48 cases of hemorrhage (46.2%, 48/104). Complete embolization was achieved in the first procedure in 64.4% of cases, and success was eventually achieved using EVT (the first attempt or a subsequent attempt) in 74.1% of cases. EVT caused complications in 6.7% of cases. A modified Rankin scale score of 0 or 1 was achieved in 78.8% of patients. Statistical analyses revealed that type Ia and IIb EVTs had the lowest complete embolization rates, but no difference was found between type Ia and IIb EVTs. Types IIa and III EVT had the highest complete embolization rates. Most cases had a good prognosis.

**Conclusion:** These findings elucidate the features of the different EVT classes defined by the first EVT procedure and the role of the MMA. The delivery of treatment via slim and tortuous MMA branches increased the failure rate of EVT. A thick, straight MMA branch is the optimal path for treatment.

## Introduction

**Figure 1:**
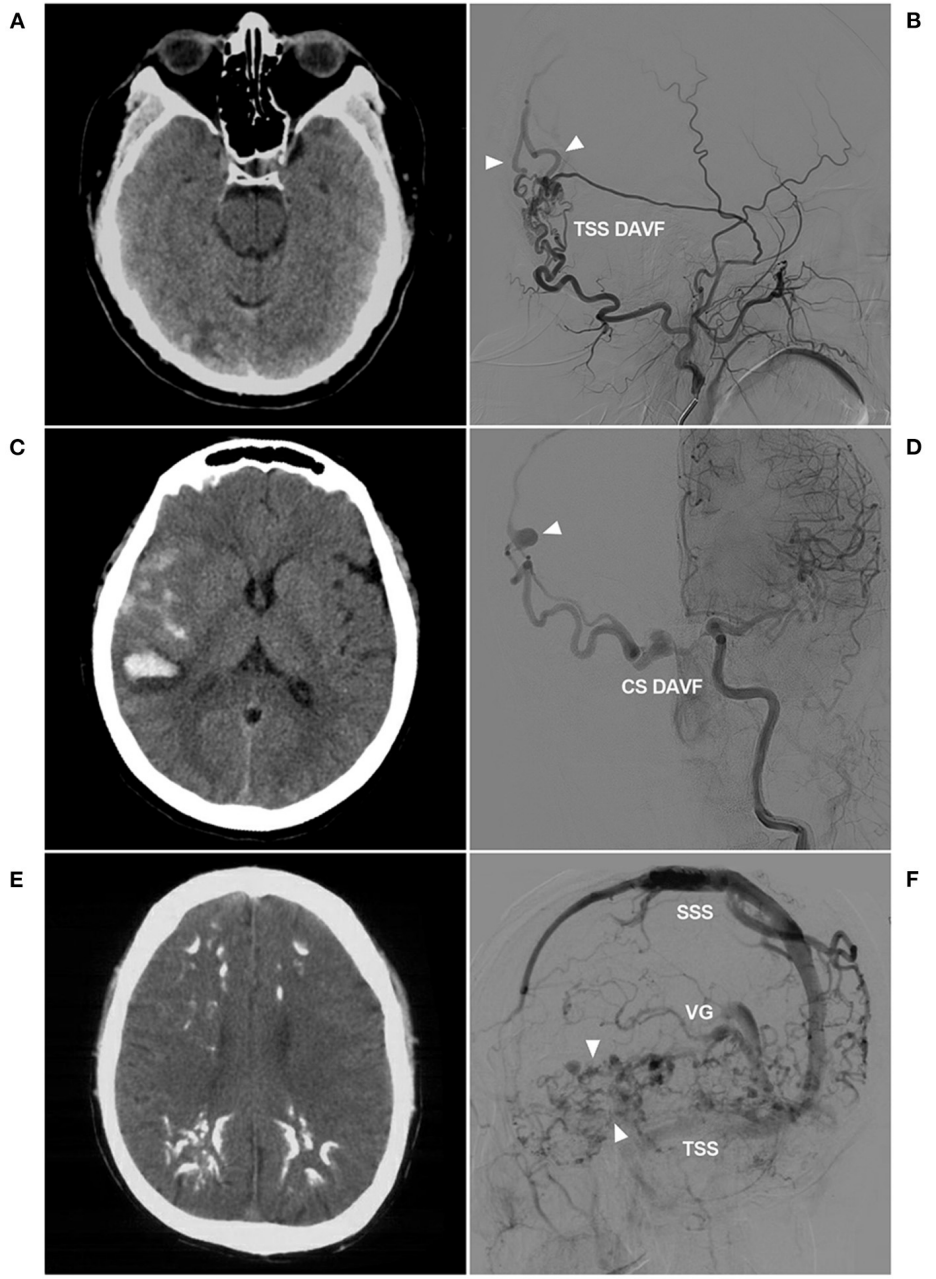
Venous drainage patterns of DAVFs. **(A,B)** Unruptured DAVF with images from the same patient. CT **(A)** showed a mix of high and low densities in the right occipital lobe. DSA **(B)** showed a TSS DAVF draining to multiple cortical veins (triangles), and the veins throughout the entire brain were normal and not dilated. **(C,D)** Hemorrhagic DAVF with images from the same patient. CT **(C)** showed subarachnoid hemorrhage and hematoma in the Sylvian fissure region. DSA **(D)** showed a CS DAVF with cortical vein drainage and a ruptured venous aneurysm (triangle). **(E,F)** Extensive pseudophlebitic DAVF with images from the same patient. CT **(E)** showed multiple calcifications in the white matter around the ventricle. DSA **(F)** showed an extensive pseudophlebitic pattern involving superficial and deep veins (triangles). CS, cavernous sinus; CT, computed tomography; DAVF, dural arteriovenous fistula; DSA, digital subtraction angiography; SSS, superior sagittal sinus; TSS, transverse-sigmoid sinus; VG, vein of Galen.

Cranial dural arteriovenous fistula (DAVF) is a rare condition that involves a direct arteriovenous connection within the dural leaflets. This condition accounts for a small proportion, ~10–15%, of all intracranial arteriovenous shunts (1). The clinical symptoms of DAVFs depend on the draining vein involved (Figure 1). DAVFs recruit many arteries to act as feeders; although the external carotid artery is usually involved, the meningeal branch of the internal carotid artery, the dural branches of the intracranial arteries, and the posterior meningeal artery (PMA, most commonly arising from the vertebral artery) may be involved (2).

**Figure 2:**
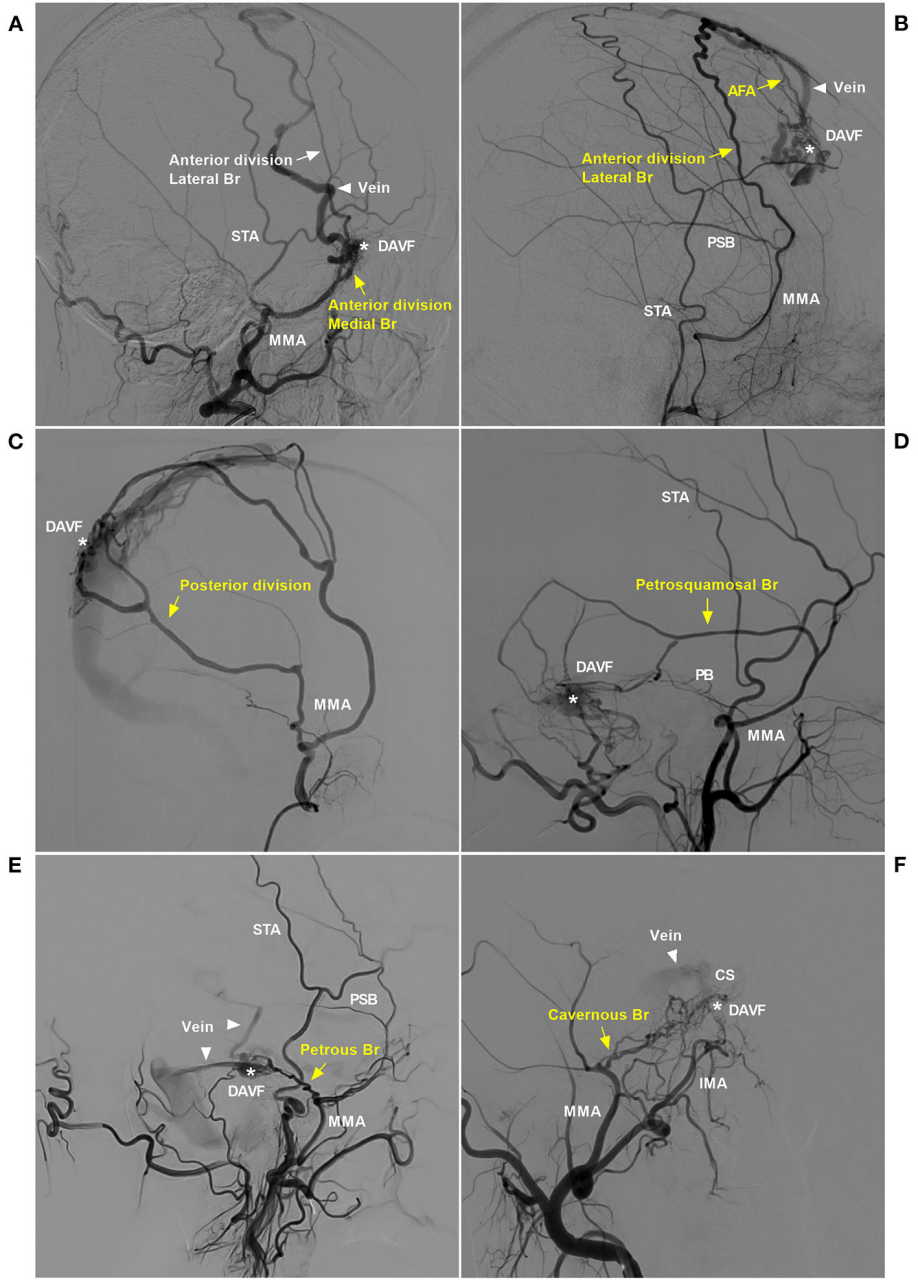
Pathological anatomy of the MMA as a DAVF feeder. **(A)** DSA of the lateral view showed the sphenoid wing DAVF (asterisk) fed by the medial branch (sphenoid) of the anterior division of the MMA (yellow arrow) draining to the SSS (triangle). **(B)** DSA of the lateral view showed a DAVF (asterisk) at the anterior falx cerebri fed by the lateral branch of the anterior division of the MMA (yellow arrow) that reached the midline and anastomosed with the AFA (yellow arrow). The DAVF drained through the cortical vein (triangle) to the SSS. **(C)** DSA of the lateral view showed that the posterior division of the MMA supplied the DAVF (asterisk) at the SSS. **(D)** DSA of the lateral view showed that the PSB of the MMA (yellow arrow) supplied the TSS DAVF (asterisk). **(E)** DSA of the lateral view showed that the PB of the MMA (yellow arrow) supplied the tentorium DAVF (asterisk), and the triangles indicate the draining veins. **(F)** DSA of the anterior posterior view showed the CS DAVF (asterisk) supplied by the cavernous branch, and the triangle indicates the draining vein. AFA, anterior falx artery; Br, branch; CS, cavernous sinus; DAVF, dural arteriovenous fistula; DSA, digital subtraction angiography; IMA, internal maxillary artery; MMA, middle meningeal artery; PB, petrous branch; PSB, petrosquamosal branch; SSS, superior sagittal sinus; STA, superficial temporal artery; TSS, transverse-sigmoid sinus.

Of all DAVF feeding arteries, the middle meningeal artery (MMA) of the external carotid artery is the most important because of its wide range of involvement, and all branches of the MMA can feed DAVFs (Figure 2). Endovascular treatment (EVT) is the primary treatment for DAVFs and focuses on the MMA (3). The first EVT procedure via the MMA determines its success or failure (4). Nevertheless, although the MMA is the gold standard arterial path of EVT for DAVFs, many factors influence the success rate (5).

Few studies have focused on the role of the MMA in EVT of DAVFs. Therefore, we performed a retrospective single-center investigation of patients who were diagnosed with a DAVF with MMA involvement.

## Materials and Methods

Patients who were admitted to the First Hospital of Jilin University and diagnosed with a cranial DAVF with MMA involvement as a feeding artery from October 2012 to October 2020 were included in this retrospective study. The institutional ethics committee approved this study.

### Inclusion and Exclusion Criteria

The inclusion criteria were as follows: (1) cranial DAVFs with feeding arteries from the MMA alone or in combination with other arteries; (2) DAVFs treated with EVT via the MMA or other arteries; and (3) no previous EVT, open surgery, or radiosurgery performed before admission to our institution. Patients with DAVFs treated with transvenous EVT were excluded.

### Strategy and Process of EVT

For cranial DAVFs with MMA as the feeding artery, digital subtraction angiography (DSA) was performed to assess the angioarchitecture of the DAVF, and the feeding artery, fistula size, draining vein, location, Cognard grade, and other parameters were recorded. When performing EVT, a Marathon or Apollo microcatheter (Medtronic, Irvine, California, USA) was used to approach the DAVF as closely as possible, and Onyx (Medtronic, Irvine, California, USA) was injected to cast the DAVF as much as possible in order to penetrate the draining vein. The EVT procedure was monitored simultaneously using angiography of another artery when necessary. If the DAVF could not be seen via the MMA and other feeding arteries, complete embolization was achieved or the EVT was incomplete.

### Classification of EVT

The present study divided EVT into three types and six subclasses based on the arterial path chosen in the first EVT procedure and the role of the MMA in EVT.

**Figure 3:**
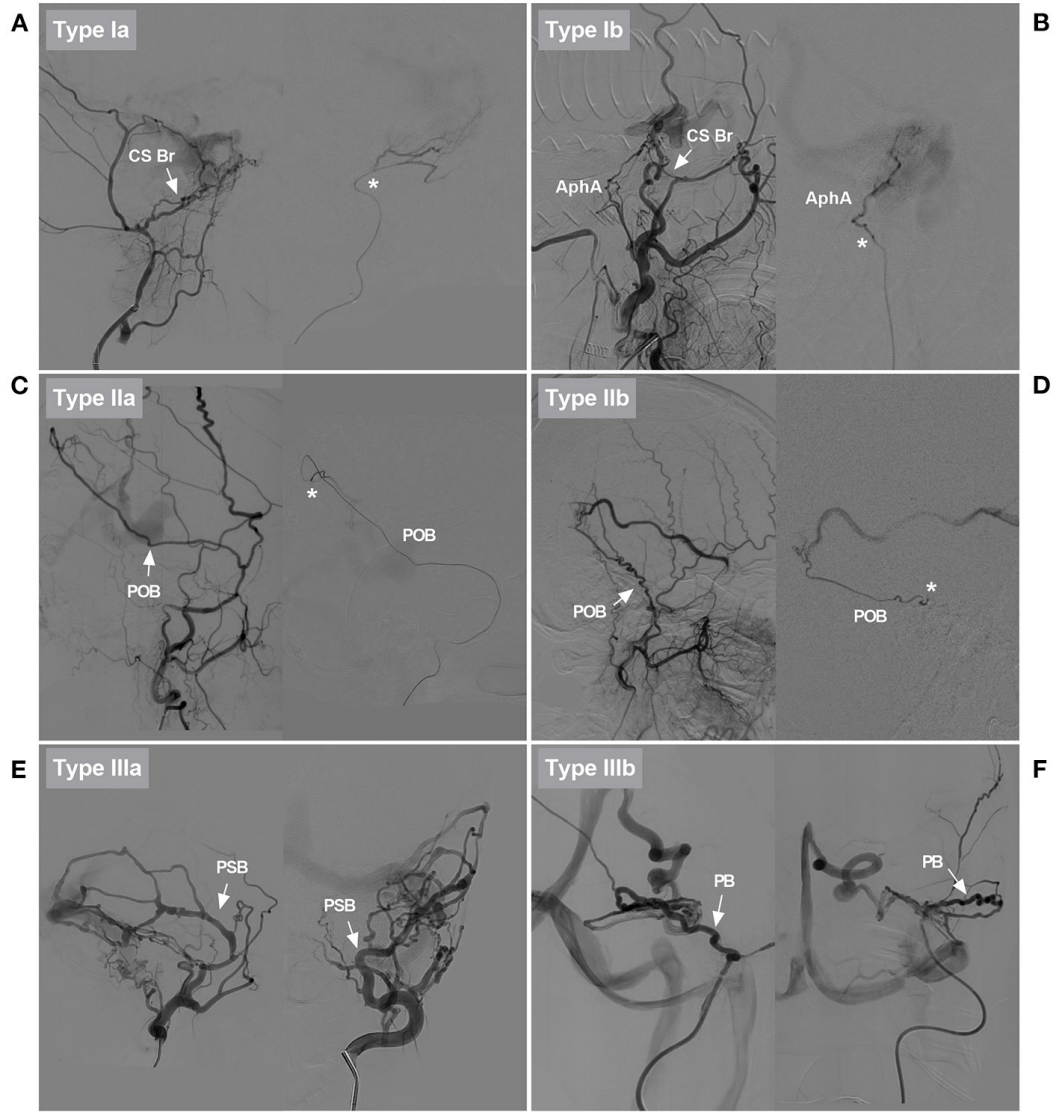
Classification of EVTs based on the role of the MMA. **(A)** DSA showed that the CS branch of the MMA in type Ia EVT was slim and tortuous (arrow). Superselective angiography showed the CS branch (asterisk). EVT was delivered via that vessel. **(B)** DSA showed that the CS branch of the MMA in type Ib EVT was slim and tortuous (arrow). EVT was delivered via the AphA, which was thicker than the CS branch. AphA was shown on superselective angiography (asterisk). **(C)** DSA showed that the POB of the MMA in type IIa EVT was normally developed, sufficiently thick, and straight (arrow). Superselective angiography showed the POB (asterisk). EVT was delivered via that vessel. **(D)** DSA showed that the PSB of the MMA in type IIb EVT was normally developed, but it was tortuous. Superselective angiography showed the PSB (asterisk). Because there was no other suitable artery, the PSB was used to deliver EVT. **(E)** Different DSA views showed that the PSB of the MMA in type IIIa EVT was overdeveloped and straight (arrows). EVT was delivered via that vessel. **(F)** Different DSA views showed that the PB of the MMA in type IIIb EVT was overdeveloped and tortuous (arrows). EVT was delivered via that vessel. AphA, ascending pharyngeal artery; Br, branch; CS, cavernous sinus; DAVF, dural arteriovenous fistula; DSA, digital subtraction angiography; EVT, endovascular treatment; MMA, middle meningeal artery; PB, petrous branch; POB, parieto-occipital branch; PSB, petrosquamosal branch.

Type I EVT: The MMA as the feeding artery was slim and tortuous, and the EVT was further divided into types a and b. In type Ia EVT, the slim and tortuous MMA branch was used to perform the embolization (Figure 3A). In type Ib EVT, a better-suited artery than the MMA was used to perform the embolization (Figure 3B).

Type II EVT: The MMA branch chosen to perform the EVT was neither slim nor normally developed, and the EVT was further divided into types a and b. In type IIa EVT, the straight MMA was used to perform the embolization (Figure 3C). In type IIb EVT, the tortuous MMA branch was used to perform the embolization (Figure 3D).

Type III EVT: The MMA branch chosen to perform EVT was overdeveloped and hyperplastic, and the EVT was further divided into types a and b. In type IIIa EVT, the straight MMA branch was used to perform the embolization (Figure 3E). In type IIIb EVT, the tortuous MMA branch was used to perform the embolization (Figure 3F).

**Figure 4:**
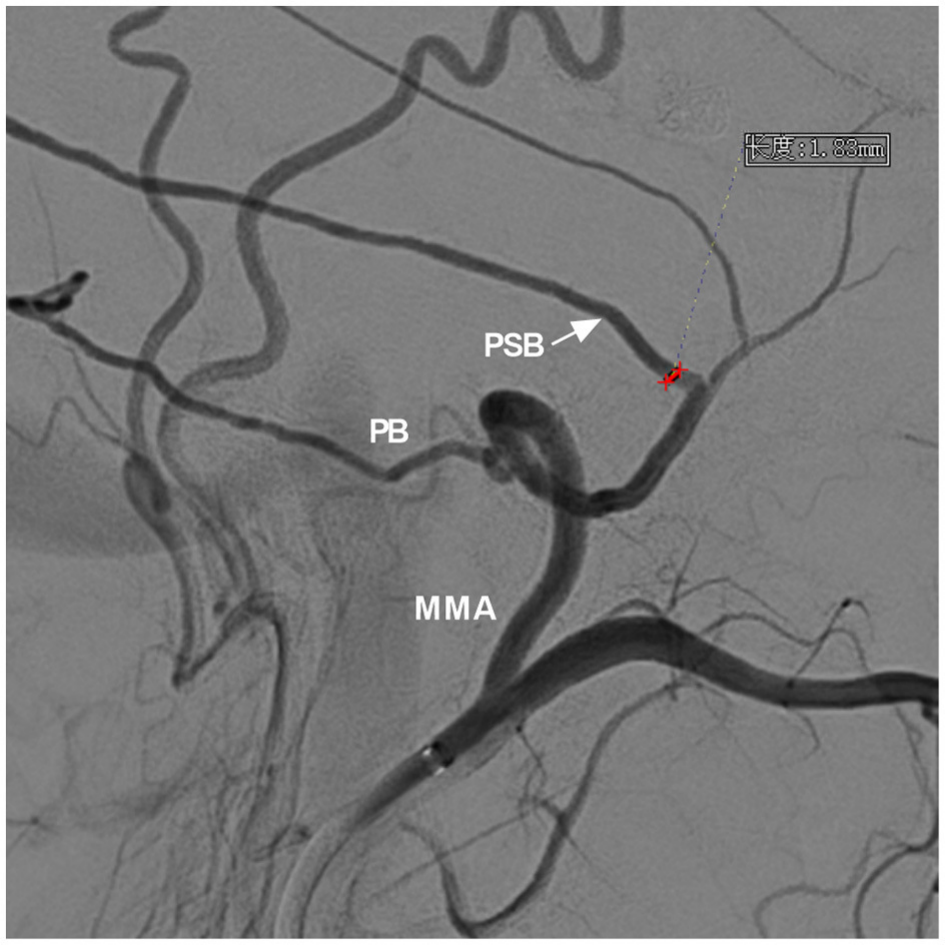
Measurement of the diameter of the MMA branch origin from the trunk. DSA showed the MMA and its branches. For the case of a DAVF with the PSB and PB acting as the main feeding arteries, the PSB (arrow) was chosen as the arterial path to perform EVT, and the diameter of the PSB origin from the trunk was measured (red line segment). DSA, digital subtraction angiography; EVT, endovascular treatment; MMA, middle meningeal artery; PB, petrous branch; PSB, petrosquamosal branch.

The diameter of the MMA branch originating from the trunk as the arterial path was measured in these types (Figure 4).

### Evaluation of Short-Term and Follow-Up Outcomes

EVT complications and resolutions, the length of hospital stay and the modified Rankin Scale (mRS) score at 3–6 months and long-term follow-up were recorded.

### Statistical Analysis

Statistical analyses were performed using GraphPad software (LLC, San Diego, CA, USA). Continuous variables are expressed as the means ± standard deviation. The chi-squared test was used to analyze count data. *P* < 0.05 was considered statistically significant.

## Results

### General Information

The 104 patients were aged 13 to 80 years (mean, 53.6 ± 11.8 years) and included 42 females (40.4%, 42/104) and 62 males (59.6%, 62/104). There were 56 cases of unruptured DAVFs (53.8%, 56/104), including 20 cases with headache and dizziness, five cases with tinnitus and intracranial murmur, seven cases with physical manifestations, 15 cases with ocular symptoms (exophthalmos and chemosis), and nine cases with cognitive deficiencies.

There were 48 cases of hemorrhage (46.2%, 48/104), including 19 cases of subarachnoid hemorrhage (SAH), 11 cases of SAH in combination with intracerebral hematoma (IH) and/or intraventricular hemorrhage (IVH), 13 cases of IH, and five cases of IH and IVH. Among the 48 cases, 17 cases were Hunt-Hess grade I, 22 cases were grade II, and nine cases were grade III.

### Imaging Characteristics

#### DAVF Location and Size

##### DAVF Location

The most common location was the transverse-sigmoid sinus (TSS) (31.7%), followed by the tentorium (21.2%), cavernous sinus (12.5%), and superior sagittal sinus (10.6%). Table 1 shows the detailed data.

**Table 1:** Locations of dural arteriovenous fistulas.

**Location**	**Number**	**Percentage**
Transverse-sigmoid sinus	33	31.7% (33/104)
Tentorium	22	21.2% (22/104)
Cavernous sinus	13	12.5% (13/104)
Superior sagittal sinus	11	10.6% (11/104)
Anterior cranial fossa	7	6.7% (7/104)
Sphenoid wing	6	5.8% (6/104)
Posterior falx cerebri	2	1.9% (2/104)
Anterior falx cerebri	2	1.9% (2/104)
Temporal region	2	1.9% (2/104)
Torcular Herophili	2	1.9% (2/104)
Middle cranial fossa	1	1.0% (1/104)
Occipital region	1	1.0% (1/104)
Parietal region	1	1.0% (1/104)
Frontal region	1	1.0% (1/104)
Total	104	100% (104/104)

##### DAVF Size

Except for six diffuse and extensive DAVFs that could not be measured, the sizes of the other 98 DAVFs were between 0.5 and 6.5 cm (mean, 3.1 ± 1.1 cm).

#### Distribution of the Feeding Artery

The feeding arteries of all 104 DAVFs included the MMA. The ipsilateral MMA was involved in 77 DAVFs (74.0%, 77/104), and the bilateral MMAs were involved in 27 DAVFs (26.0%, 27/104). The other feeding arteries were the occipital artery (OA) (47.1%), PMA (29.8%), and meningohypophyseal trunk (MHT) (26.0%). Table 2 shows the detailed data.

**Table 2:** Distribution of feeding arteries of dural arteriovenous fistulas.

**Artery**	**Number**	**Percentage**
Middle meningeal artery	104	100% (104/104)
Occipital artery	49	47.1% (49/104)
Posterior meningeal artery	31	29.8% (31/104)
Meningohypophyseal trunk	27	26.0% (27/104)
Inferior lateral trunk	12	11.5% (12/104)
Ascending pharyngeal artery	12	11.5% (12/104)
Ophthalmic artery	10	9.6% (10/104)
Posterior cerebral artery	9	8.7% (9/104)
Accessory meningeal artery	6	5.8% (6/104)
Superficial temporal artery	5	4.8% (5/104)
Middle cerebral artery	5	4.8% (5/104)
Anterior inferior cerebellar artery	3	2.9% (3/104)
Superior cerebellar artery	3	2.9% (3/104)

#### Venous Drainage Pattern

Multiple cortical veins providing drainage were the most common pattern in the DAVFs (33.7%), followed by a single cortical vein (19.2%), a single deep vein (9.6%), multiple deep veins (9.6%), and the vein of Galen with reverse flow (4.8%). Table 3 shows the detailed data.

**Table 3:** Venous drainage patterns of dural arteriovenous fistulas.

**Draining vein**	**Number**	**Percentage**
Multiple cortical veins	35	33.7% (7/104)
Single cortical vein	20	19.2% (20/104)
Venous sinus or ophthalmic vein	13	12.5% (14/104)
Single deep vein	10	9.6% (10/104)
Multiple deep veins	10	9.6% (10/104)
Both cortical and deep veins	7	6.7% (7/104)
Vein of Galen with reverse flow	5	4.8% (5/104)
Spinal cord vein	4	3.8% (4/104)
Total	104	100% (104/104)

#### Cognard Grade of DAVFs

Four of the 104 DAVFs were Cognard grade I (3.8%, 4/104), five DAVFs were grade IIa (4.8%, 5/104), two DAVFs were grade IIb (1.9%, 2/104), 22 DAVFs were grade IIa+b (21.2%, 22/104), 10 DAVFs were grade III (9.6%, 10/104), 57 DAVFs were grade IV (54.8%, 57/104), and four DAVFs were grade V (3.8%, 4/104).

#### EVT Classification and Choice of the Arterial Path

##### EVT Classification

Based on the arterial path chosen in the first EVT procedure, type Ia EVT was performed for 14 DAVFs (13.5%, 14/104), type Ib EVT was performed for 16 DAVFs (15.4%, 16/104), type IIa EVT was performed for 48 DAVFs (46.1%, 48/104), type IIb EVT was performed for nine DAVFs (8.7%, 9/104), type IIIa EVT was performed for 11 DAVFs (10.6%, 11/104), and type IIIb EVT was performed for six DAVFs (5.8%, 6/104).

The baseline data of patients were compared among types I, II, and III EVT. There was no difference in age, sex, or onset (Table 4).

**Table 4:** Baseline data of type I, II, and III EVTs.

	**I (*n* = 30)**	**II (*n* = 57)**	**III (*n* = 17)**	***P-*value**
Age (years)	59.1 ± 9.1	52.0 ± 12.3	49.4 ± 11.5	0.2146 (Bartlett's test)
Sex (male)	16	34	12	0.5112 (Chi-square, 1.342)
Nonhemorrhagic onset	15	31	10	0.8375 (Chi-square, 0.356)

*EVT, endovascular treatment*.

##### Choice of the Arterial Path

Among the 104 first EVT procedures, MMA branches were used in 88 of them (84.6%, 88/104), most commonly the petrosquamosal branch (PSB) (34.6%), followed by the parieto-occipital branch (POB) (14.4%), cavernous sinus branch (10.6%), and petrous branch (9.6%). Type Ib EVT was performed via the OA in 16 cases (8.5%), followed by the ascending pharyngeal artery (AphA) (2.9%) and ophthalmic artery (OphA) (1.9%). Table 5 shows the detailed data.

**Table 5:** Arterial path used in the first EVT procedure.

**Arterial path**	**Number**	**Percentage**
Petrosquamosal branch	36	34.6% (36/104)
Parieto-occipital branch	15	14.4% (15/104)
Petrous branch	10	9.6% (10/104)
Cavernous sinus branch	11	10.6% (11/104)
Medial branch of anterior division	7	6.7% (7/104)
Lateral branch of anterior division	6	5.8% (6/104)
Accessory meningeal artery	3	2.9% (3/104)
Occipital artery	9	8.5% (9/104)
Ascending pharyngeal artery	3	2.9% (3/104)
Ophthalmic artery	2	1.9% (2/104)
Internal maxillary artery	2	1.9% (2/104)
Total	104	100% (104/104)

*EVT, endovascular treatment*.

The diameter of the MMA branch originating from the trunk as the arterial path was obtained only in a few cases: type I (0.5 ± 0.1 mm, *n* = 16); type II (1.8 ± 0.2 mm, *n* = 26); and type III (2.7 ± 0.4 mm, *n* = 8).

### EVT Result and Subsequent Processing

Complete embolization was obtained in 67 cases after the first EVT procedure (64.4%, 67/104), and incomplete embolization was obtained in 37 cases (35.6%, 37/104). Among the 37 cases of unsuccessful EVT, Onyx did not reach the fistula point in two cases, and it reached the fistula but did not embolize the DAVF completely in 35 cases.

Among the 37 cases of unsuccessful EVT, temporary conservative treatment was used in 17 cases, and direct surgical resection was performed in four cases. EVT via other arteries was performed in the other 16 cases. Complete EVT was achieved in 10 cases, and conservative treatment was administered in the other six cases.

In summary, the first EVT procedure achieved complete embolization with a success rate of 64.4% (67/104), and the success rate for the first and subsequent EVT procedures was 74.1% (77/104).

The baseline data were compared between the incomplete and complete EVT groups, and there was no difference in age, sex, or onset (Table 6).

**Table 6:** Baseline data in patients with incomplete and complete EVTs.

	**Incomplete EVT**	**Complete EVT**	***P-*value**
	**(*n* = 37)**	**(*n* = 67)**	
Age (years)	54.7 ± 12.7	53.1 ± 11.3	0.5172 (Unpaired *t-*test)
Sex (male)	18	44	0.0998 (Fisher's exact test)
Nonhemorrhagic	25	31	0.0631 (Fisher's exact test)

*EVT, endovascular treatment*.

### Complications and Solutions

EVT resulted in complications in seven cases (6.7%, 7/104). Facial numbness was observed in two cases, and conservative treatment was administered. Intraoperative or postoperative intracranial hemorrhage occurred in three cases. Hematoma evacuation was applied in two cases, and conservative treatment was administered in one case. Blindness occurred in one case, and conservative treatment was administered. Postoperative hydrocephalus occurred in one case, and a ventriculoperitoneal shunt was performed.

### Follow-Up and Outcomes

The length of hospital stay ranged from 1 to 20 days (4.2 ± 3.5 days).

The short-term follow-up period ranged from 3 to 6 months. The mRS scores were 0, 1, 2, and 3–5 in 56 (53.8%, 56/104), 26 (25%, 26/104), 18 (17.3%, 18/104), and 4 (3.8%, 4/104) patients, respectively. Good short-term recovery (mRS score of 0 or 1) was achieved in 78.8% (82/104) of the patients.

The long-term follow-up period ranged from 9 to 102 months. All 59 patients with telephone follow-up data had a mRS score of 0 or 1. Only 31 patients had angiographic follow-up data. Twenty-two patients had no recurrence, and nine patients had incomplete embolization and accepted retreatment.

### Statistical Analysis

The relationship between the EVT degree and the artery chosen as the path of the first EVT procedure was analyzed. After excluding six diffuse and extensive DAVFs, 98 DAVFs with first EVT attempts were included in the statistical analysis.

The chi-squared test was used to identify differences between multiple groups. Types Ia and IIb EVTs had the lowest complete embolization rates, but there was no difference between types Ia and IIb EVTs. Types IIa and III had the highest complete embolization rates. Tables 7, 8 show the detailed data.

**Table 7:** Statistical analyses of EVT completion.

**EVT type**	**Complete EVT**	**Incomplete EVT**	**Total**	
Ia	2 (14.3%)	12 (85.7%)	14	Chi-squared, 35.4, *P* < 0.0001
Ib	10 (62.5%)	6 (37.5%)	16	
IIa	40 (87.0%)	6 (13.0%)	46	
IIb	3 (33.3%)	6 (66.7%)	9	
IIIa	6 (85.7%)	1 (14.3%)	7	
IIIb	6 (100%)	0 (0%)	6	
Total	67 (68.4%)	31 (31.6%)	98	

*Chi-squared = 35.4, P < 0.01, indicating a difference between the six subgroups*.

*EVT, endovascular treatment*.

**Table 8:** Statistical comparisons between type Ia or IIb EVTs and other EVTs.

**EVT type**	**Ia**	**IIb**
Ia	NA	*P* = 0.3428
Ib	*P* = 0.0106	*P* = 0.2262
IIa	*P* <0.0001	*P* = 0.0019
IIb	*P* = 0.3428	NA
III	*P* <0.0001	*P* = 0.0066

*Fisher's exact test was performed, and the analysis showed that type Ia and IIb EVTs had the lowest complete embolization rates but that there was no difference between type Ia and IIb EVTs. Types IIa and III EVT had the highest complete embolization rates*.

*EVT, endovascular treatment. NA, not applicable*.

**Figure 5:**
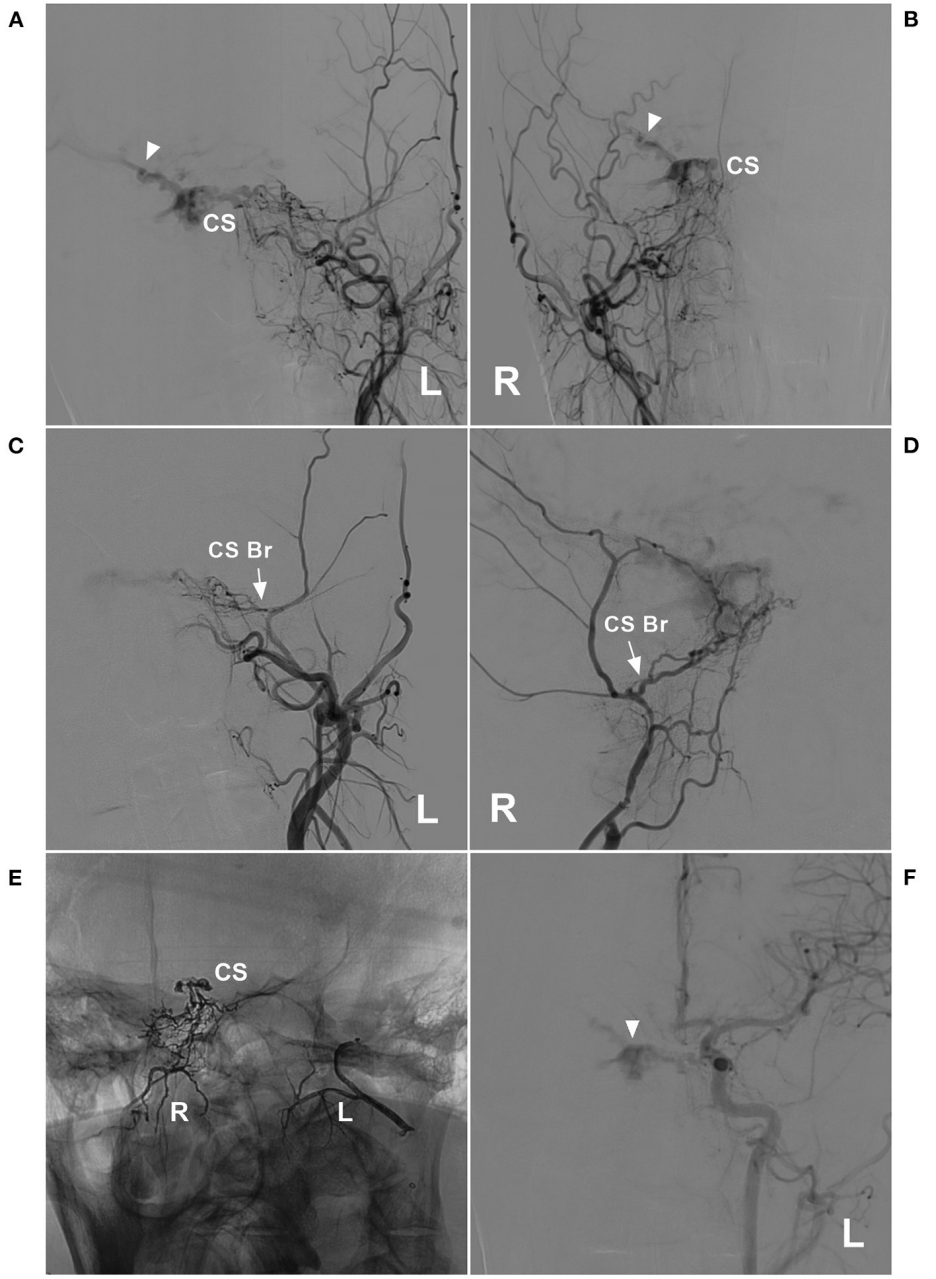
Unsuccessful type Ia EVT of a CS DAVF. **(A,B)**: Left **(A)** and right **(B)** DSA of the anterior posterior view showed a CS DAVF with cortical vein drainage in the Sylvian fissure (triangles). **(C,D)** Left **(C)** and right **(D)** DSA showed the CS branch supplying the DAVF (arrows). **(E)** X-ray film showed bilateral embolization with Onyx. Onyx was successfully injected into the CS. **(F)** DSA of the left carotid artery showed that the EVT was incomplete, and the DAVF remained visible (triangle). Br, branch; CS, cavernous sinus; DAVF, dural arteriovenous fistula; DSA, digital subtraction angiography; EVT, endovascular treatment; L, left; R, right.

**Figure 6:**
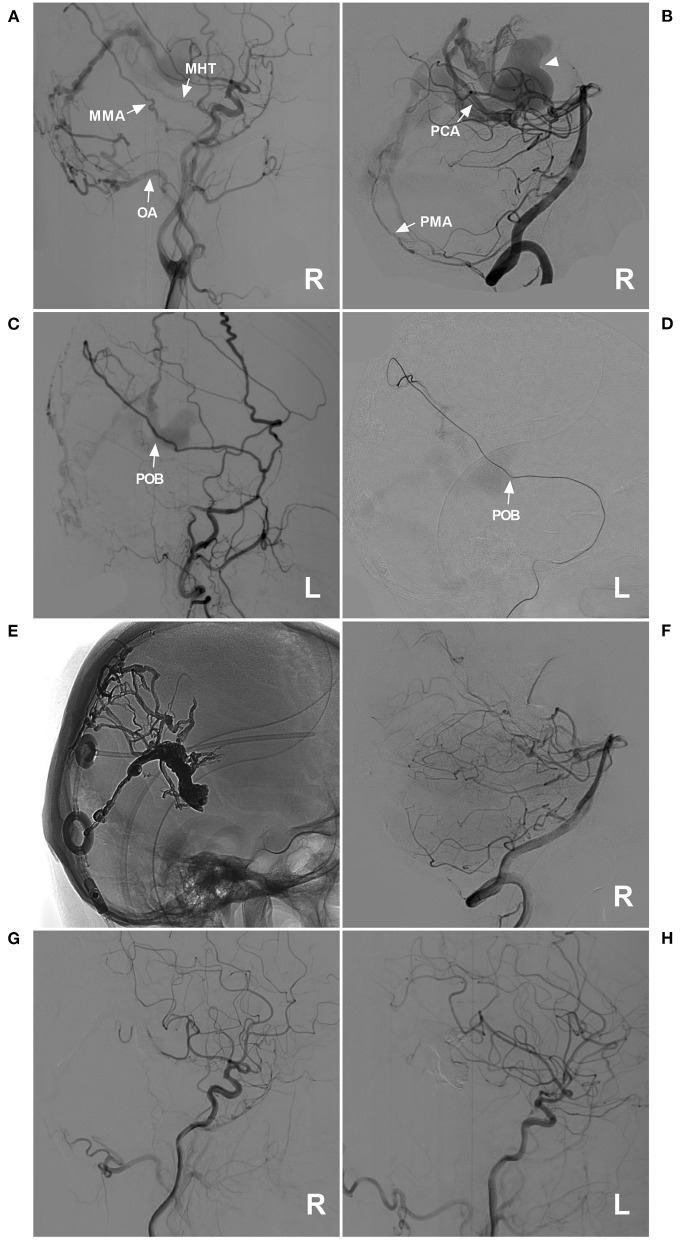
Successful type IIa EVT for a DAVF of the posterior falx cerebri. **(A)** Lateral view DSA of the right carotid artery showed a DAVF at the posterior falx cerebri that was supplied by the MHT, the POB of the MMA, and the OA (arrows). **(B)** DSA of the right VA showed that the dural branch of the PCA (arrow) and the PMA (arrow) supplied the DAVF. The dilated draining vein is indicated by a triangle. **(C,D)**: Lateral view DSA of the left ECA **(C)** and superselective angiography of the POB **(D)** showed that the POB was normally developed and straight (arrows). **(E)** X-ray film showing the Onyx casting and previously established ventriculoperitoneal shunt. **(F,H)** Images of the right VA **(F)**, right carotid artery **(G)**, and left carotid artery **(H)** showed that the EVT was complete. DAVF, dural arteriovenous fistula; DSA, digital subtraction angiography; ECA, external carotid artery; EVT, endovascular treatment; L, left; MHT, meningohypophyseal trunk; MMA, middle meningeal artery; OA, occipital artery; PCA, posterior cerebral artery; PMA, posterior meningeal artery; POB, parieto-occipital branch; R, right; VA, vertebral artery.

**Figure 7:**
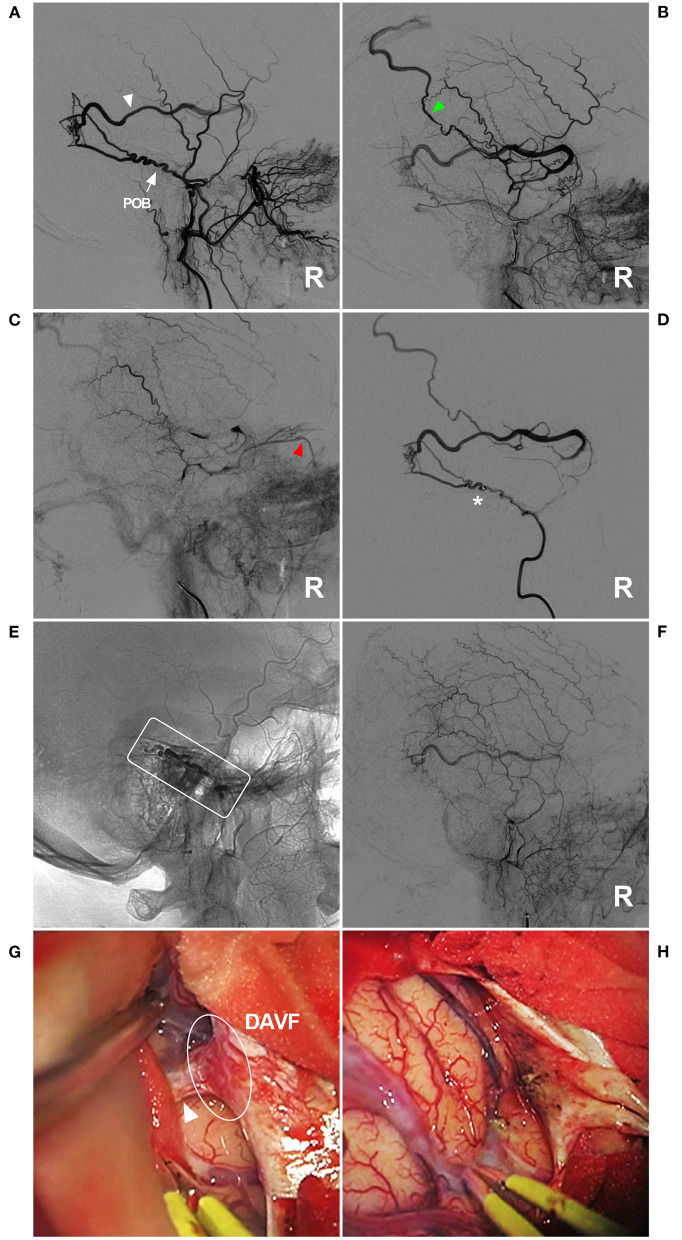
Unsuccessful type IIb EVT for a DAVF in the temporal region. **(A–C)** Lateral view ECA DSA showed a DAVF in the temporal region supplied by a normally developed and tortuous POB (arrow) **(A)**. The draining vein behind the Sylvian fissure is clearly shown [white triangle in panel **(A)**, early arterial phase] draining to the superior sagittal sinus [green triangle in panel **(B)**, early arterial phase] and the ophthalmic vein [red triangle in panel **(C)**, late arterial phase]. **(D)** Right MMA angiography showed that the microcatheter could not pass through the tortuous POB (asterisk). **(E)** X-ray film showing that Onyx (frame) could not reach the point of the fistula. **(F)** DSA showing the incomplete EVT. **(G)** Intraoperative imaging showing the DAVF (ellipse) and the draining vein (triangle). **(H)** The DAVF and draining vein were coagulated and cut. DAVF, dural arteriovenous fistula; DSA, digital subtraction angiography; ECA, external carotid artery; EVT, endovascular treatment; MMA, middle meningeal artery; POB, parieto-occipital branch; R, right.

**Figure 8:**
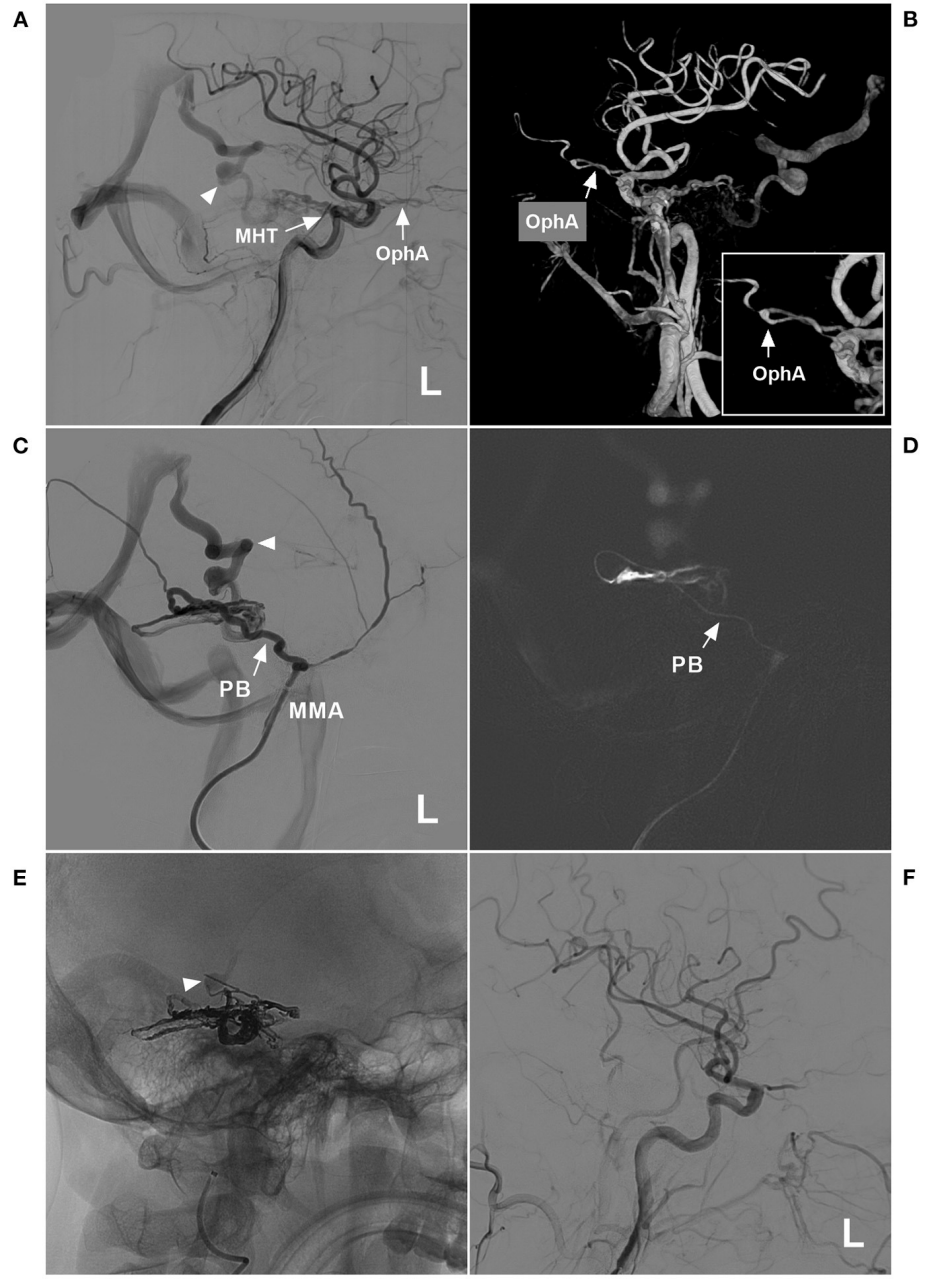
Successful type IIIb EVT for a DAVF of the tentorium. **(A)** Lateral view DSA of the left carotid artery showed a DAVF supplied by the MHT and OphA (arrows) draining to the vein of Galen (triangle). **(B)** Three-dimensional DSA showed that the recurrent meningeal branch of the OphA supplied the DAVF (arrow). The inset in the lower right shows the magnified local OphA (arrow). **(C)** Left MMA angiography showed that the PB of the MMA supplied the DAVF. The triangle indicates deep vein drainage. **(D)** A roadmap image shows that the microcatheter passed through the PB (arrow) and into the fistula. **(E)** X-ray film showing the casting Onyx. Onyx penetrated the fistula and sealed the draining vein (triangle). **(F)** The left carotid artery showed that the EVT was complete. DAVF, dural arteriovenous fistula; DSA, digital subtraction angiography; EVT, endovascular treatment; L, left; MHT, meningohypophyseal trunk; MMA, middle meningeal artery; OphA, ophthalmic artery; PB, petrous branch.

**Figure 9:**
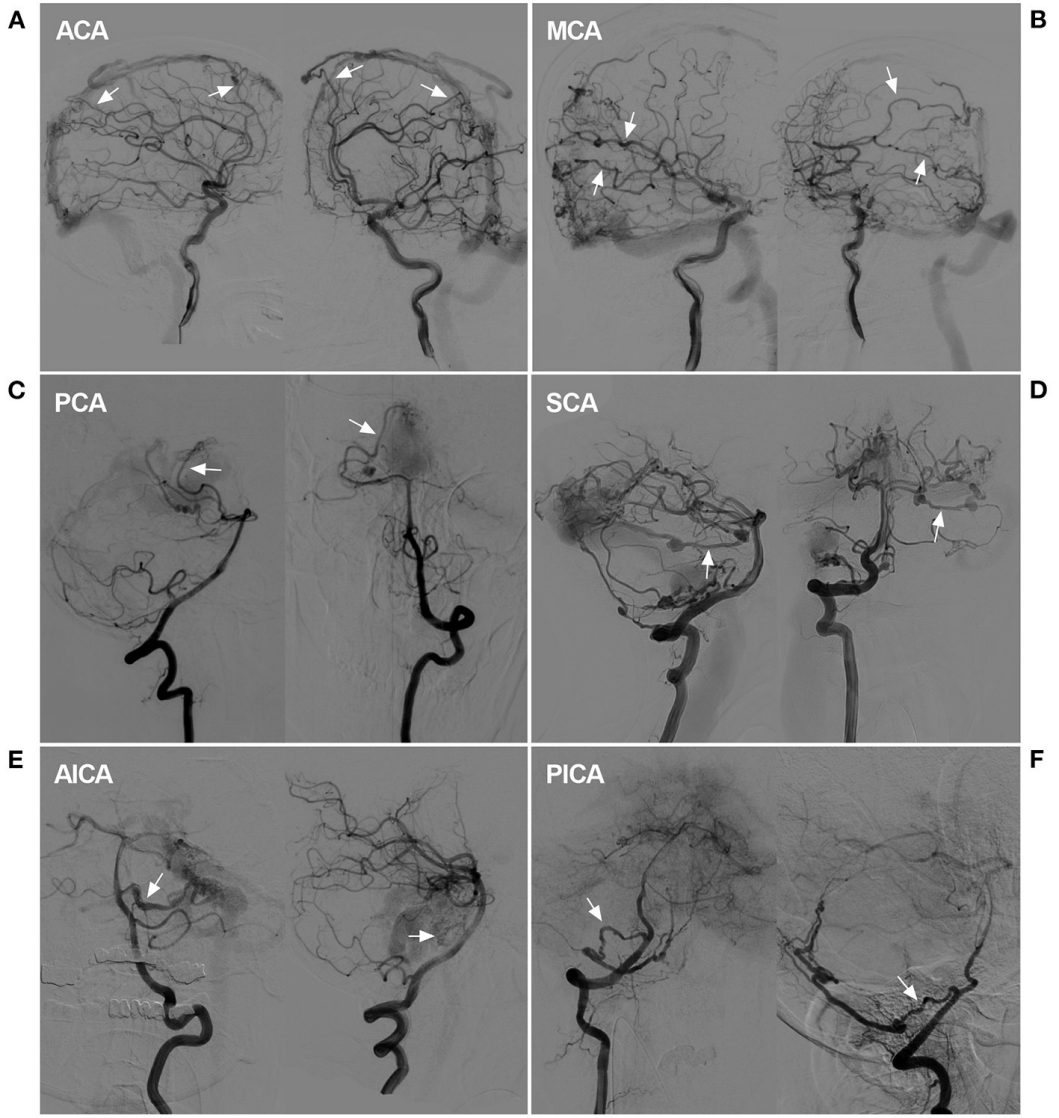
Rare instance of a pial artery serving as the feeder for a DAVF. **(A)** DSA showed that the ACA sent dural branches (arrows) to supply a SSS DAVF. **(B)** DSA showed that the MCA sent dural branches (arrows) to supply a SSS DAVF. **(C)** DSA showed that the PCA (arrows) sent a dural branch to supply a tentorial DAVF. **(D)** DSA showed that the SCA (arrows) sent a dural branch to supply a TSS DAVF. A flow-related aneurysm may be seen. **(E)** DSA showed that the AICA (arrows) sent a dural branch to supply a tentorial DAVF. A dissecting aneurysm may be seen. **(F)** DSA showed that the PICA (arrows) connected to the PMA to supply a tentorial DAVF. ACA, anterior cerebral artery, AICA, anterior inferior cerebellar artery; DAVF, dural arteriovenous fistula; DSA, digital subtraction angiography; MCA, middle cerebral artery, PCA, posterior cerebral artery, PICA, posterior inferior cerebellar artery; PMA, posterior meningeal artery; SCA, superior cerebellar artery; SSS, superior sagittal sinus; TSS, transverse-sigmoid sinus.

We also demonstrated some typical and educational cases of EVT (Figures 5–**9**).

## Discussion

A DAVF is an arteriovenous shunt located in the dura. The current treatment is EVT, which can involve transarterial and transvenous approaches (6). Although the transvenous approach is an effective method for treating some DAVFs, such as EVT via the cavernous sinus and certain TSSs, the transarterial approach remains the primary approach for treating most DAVFs (7).

Most of the feeding arteries may be used for the arterial path, but the MMA always plays the most important role. Indeed, the MMA is the gold standard artery for the transarterial approach for DAVFs (8). However, few studies have examined the role and influencing factors of the MMA in EVT. Our study collected data from 104 DAVFs with MMA involvement as the feeding artery for analysis of the treatment success rate.

The MMA is widely distributed throughout the cranium, which results in most DAVFs using the MMA as the feeding artery (9). In our study of DAVFs with MMA involvement, the most common location was the TSS, followed by the tentorium and cavernous sinus, at rates of 31.7, 21.2, and 12.5%, respectively. The reason for this finding is that the branches of the MMA easily reach these locations, which tend to exhibit DAVFs. For cranial DAVFs, the MMA is the most common source as the feeding artery (10). Certainly, other arteries may be involved, including the OA, PMA, and MHT, which occurred at rates of 47.1, 29.8, and 26.0%, respectively. These arteries tend to supply the DAVF, but this tendency is because DAVFs of the TSS and tentorium were more common in our study.

In general, the symptoms of DAVFs are divided into hemorrhagic and nonhemorrhagic types (10). In our study of 104 DAVFs, 46.2% of patients had hemorrhagic symptoms at onset. The hemorrhagic onset was primarily from cortical and deep vein drainage (1). The most common pattern of drainage was via multiple cortical veins, with a rate of 33.7%.

The symptoms of DAVFs depended on the pattern of venous drainage, as shown in Figure 1A. Although the arterial blood was diverted to a local vein in some cases, the vein was unruptured, and there were fewer or no symptoms. Figure 2B shows a ruptured venous aneurysm, and this patient presented with hemorrhagic symptoms. Although the veins were not ruptured in Figure 2C, the arterial blood was diverted into the whole brain vein via the vein of Galen with reverse flow. This pattern is called an extensive pseudophlebitic pattern, and the patient presented with cognitive deficiencies (11). The extensive pseudophlebitic pattern occurred at a rate of 4.8% in our study, and Cognard grade IV accounted for 54.8% of these cases, which corresponded to the hemorrhagic presentation.

Our study involves an in-depth evaluation of the role of MMA in the transarterial EVT of 98 DAVFs after the exclusion of six diffuse DAVFs. Most of the arteries throughout the entire cranium were recruited in these cases of extensive and diffuse DAVFs, including the bilateral arteries and dural branches of intracranial arteries (Figure 9) and the posterior cerebral artery (the artery of Davidoff and Schechter), superior cerebellar artery (the artery of Wollschlaeger and Wollschlaeger), anterior inferior cerebellar artery and posterior inferior cerebellar artery (12, 13).

It is apparent that EVT via these dural branches of the intracranial arteries is difficult and dangerous (14). Therefore, transarterial EVT must depend more on the MMA (15). The common branches in our study included the PSB, POB, petrosal branch, cavernous branch, lateral branch and medial branch (sphenoid) of the anterior division. Under normal conditions, these vessels are slim or invisible, but they become thicker and overdeveloped under the pathological condition of serving as feeding arteries of a DAVF (Figure 2).

We treated DAVFs via the MMA and found that some DAVFs could be completely embolized but that other DAVFs could not, and we wanted to know the underlying reason for this difference. The first path chosen for EVT is very important and key to successful EVT. Therefore, the MMA branch must be evaluated carefully. Our study design considered thickness and tortuosity to be the most influential factors. Therefore, we divided EVT into three types and six subclasses (Figure 3).

Statistical analyses showed that type Ia and IIb EVTs had the lowest complete embolization rates, and type IIa and III EVTs had the highest complete embolization rates. This result means that a thicker and straighter MMA branch is the preferred path. If only types IIa and III EVTs are performed, the success rate may be near 90%. EVT for DAVFs with MMA involvement generally resulted in a good prognosis, and mRS scores of 0 and 1 were achieved in 78.8% of patients. However, EVT resulted in complications in 6.7% of patients.

Complications included facial numbness in two cases, blindness in one case, intraoperative or postoperative intracranial hemorrhage in three cases, and postoperative hydrocephalus in one case. Facial numbness, as a form of cranial nerve damage, resulted from the dangerous anastomosis of the MMA, in which the liquid material enters the feeder to affect the geniculate ganglion (16). In the case of blindness, the liquid material entered the ophthalmic artery via the MMA (17). If the fistula ruptures during EVT under the high pressure of Onyx casting or if too many draining veins are occluded, intracranial hemorrhage may occur. Therefore, EVT must be performed carefully and gently to prevent damage to the draining veins (18). If the draining veins are too thick and near the aqueduct of the midbrain, thrombosis in the draining veins after EVT may cause hydrocephalus (19), and a cerebrospinal fluid shunt is needed.

## Conclusions

The findings of this study elucidated the features of different EVT classes as defined by the first EVT procedure and the role of the MMA. The delivery of treatment via slim and tortuous MMA branches increased the failure rate of EVT. A thick, straight MMA branch is the optimal path for treatment. EVT for DAVFs with MMA involvement generally results in a good prognosis.

### Limitations

This was a retrospective study, and its conclusions should be interpreted cautiously. As a result of the economic status in rural areas in China, angiographic follow-up data were challenging to obtain, which made it difficult to evaluate the long-term efficacy of EVT. The short-term follow-up in this study was complete, and we performed long-term telephone follow-up in April 2021. Only 50% of patients had clinical follow-up data, and 20% of patients had imaging follow-up data. There was an inherent flaw in the measurement of the MMA diameter because it is impossible to accurately obtain the entire course, and only the origin of the MMA branch from the trunk was measured as the EVT path. Due to the retrospective nature of the study, these data were obtained only for some patients, which reduces the clinical importance of these data. However, our analyses of the arterial path used in the first EVT procedure and the role of the MMA in EVT are of the utmost importance.

## Data Availability Statement

The original contributions presented in the study are included in the article/supplementary material, further inquiries can be directed to the corresponding author/s.

## Ethics Statement

The studies involving human participants were reviewed and approved by the First Hospital of Jilin University. The patients/participants provided their written informed consent to participate in this study. Written informed consent was obtained from the individual(s) for the publication of any potentially identifiable images or data included in this article.

## Author Contributions

JY and KX: contributed to the conception and design of the manuscript and critically revised the manuscript. HS and YW: wrote the manuscript. HS: collected the medical records of the patients. All authors approved the final version of this manuscript.

## Conflict of Interest

The authors declare that the research was conducted in the absence of any commercial or financial relationships that could be construed as a potential conflict of interest.
